# Supplementary material for: The Systems Biology Research Tool: evolvable open-source software
Source: BMC Syst Biol. 2008 Jun 29;2:55. doi: 10.1186/1752-0509-2-55 (PMC2446383; doi:10.1186/1752-0509-2-55)
Supplement: Additional file 1 — SBRT Archive. An archive of the current version of the Systems Biology Research Tool. [file 1752-0509-2-55-S1.zip › sbrt-1.4.0/doc/developers_guide/api/sbrt/kernel/math/algebra/AbstractMdMatrix.html]

AbstractMdMatrix


|  |  |  |
| --- | --- | --- |
| **Overview** \| **Package** \| **Class** \| **Use** \| **Tree** \| **Deprecated** \| **Index** \| **Help** | |  |
| PREV CLASS   **NEXT CLASS** | **FRAMES**    **NO FRAMES**     **All Classes** |  |
| SUMMARY: NESTED \| FIELD \| CONSTR \| METHOD | DETAIL: FIELD \| CONSTR \| METHOD |  |


---


## sbrt.kernel.math.algebra Class AbstractMdMatrix<R,C>

```
java.lang.Object
  sbrt.kernel.math.algebra.AbstractMdMatrix<R,C>
```

**Type Parameters:**: `R` - the row type.: `C` - the column type.

**All Implemented Interfaces:**: DoubleMatrix<R,C>, DoubleMatrixBuilder<R,C>, Matrix<R,C,java.lang.Double>, MatrixBuilder<R,C,java.lang.Double>

**Direct Known Subclasses:**: AbstractSmdMatrix

---

``` public abstract class AbstractMdMatrix<R,C> extends java.lang.Object implements DoubleMatrixBuilder<R,C> ```

This abstract class is a skeleton implementation of
`DoubleMatrixBuilder`.

**Author:**
:   This class was written and documented by
    Jeremiah Wright while in the Wagner lab.

---

**Constructor Summary**

`AbstractMdMatrix()`


| **Method Summary** | |
| --- | --- |
| `boolean` | `equals(java.lang.Object obj)`             Indicates if the provided object is equal to this matrix. |
| `boolean` | `isEmpty()`             Indicates if this matrix is empty. |
| `void` | `setElements(DoubleMatrix<? extends R,? extends C> m)`             Sets the corresponding elements of this matrix to those of the provided matrix. |
| `java.lang.String` | `toString()`             Returns a string representation of this matrix. |

**Methods inherited from class java.lang.Object**

`clone, finalize, getClass, hashCode, notify, notifyAll, wait, wait, wait`

**Methods inherited from interface sbrt.kernel.math.algebra.DoubleMatrixBuilder**

`setElement`

**Methods inherited from interface sbrt.kernel.math.algebra.MatrixBuilder**

`removeColumn, removeRow, setElement, setElements`

**Methods inherited from interface sbrt.kernel.math.algebra.Matrix**

`columns, getColumnIndices, getRowIndices, hashCode, isColumnIndex, isRowIndex, rows`

**Methods inherited from interface sbrt.kernel.math.algebra.DoubleMatrix**

`getColumn, getElement, getRow, toLinearCombs`

**Methods inherited from interface sbrt.kernel.math.algebra.Matrix**

`columns, getColumnIndices, getRowIndices, hashCode, isColumnIndex, isRowIndex, rows`

**Constructor Detail**

### AbstractMdMatrix

```
public AbstractMdMatrix()
```


**Method Detail**

### isEmpty

```
public boolean isEmpty()
```

:   Indicates if this matrix is empty.

    :   **Specified by:**: `isEmpty` in interface `Matrix<R,C,java.lang.Double>`
    :   **Returns:**: `rows() == 0 && columns() == 0`.

---


### toString

```
public java.lang.String toString()
```

:   Returns a string representation of this matrix.

    :   **Specified by:**: `toString` in interface `Matrix<R,C,java.lang.Double>` **Overrides:**: `toString` in class `java.lang.Object`
    :   **Returns:**: a string representation of this matrix.

---


### equals

```
public boolean equals(java.lang.Object obj)
```

:   Indicates if the provided object is equal to this matrix.
    Two matrices are considered equal if they share the same
    sets of column and row indices and the elements in each
    matrix for each column-row index pair are the same.

    :   **Specified by:**: `equals` in interface `Matrix<R,C,java.lang.Double>` **Overrides:**: `equals` in class `java.lang.Object`
    :   **Parameters:**: `obj` - the object with which to compare. **Returns:**: `true` if this object equals the provided object; `false` otherwise.

---


### setElements

```
public void setElements(DoubleMatrix<? extends R,? extends C> m)
```

:   Sets the corresponding elements of this matrix to
    those of the provided matrix.

    :   **Specified by:**: `setElements` in interface `DoubleMatrixBuilder<R,C>`
    :   **Parameters:**: `m` - the matrix whose elements are to be copied into this matrix.


---


|  |  |  |
| --- | --- | --- |
| **Overview** \| **Package** \| **Class** \| **Use** \| **Tree** \| **Deprecated** \| **Index** \| **Help** | |  |
| PREV CLASS   **NEXT CLASS** | **FRAMES**    **NO FRAMES**     **All Classes** |  |
| SUMMARY: NESTED \| FIELD \| CONSTR \| METHOD | DETAIL: FIELD \| CONSTR \| METHOD |  |


---
